# Perinatal Health Promotion in Indigenous Maternal Health: A Scoping Review of Peer-Reviewed Evidence and Australian Community-Controlled Programs

**DOI:** 10.3390/ijerph23060746

**Published:** 2026-06-02

**Authors:** Cecilia Castiello, Kai W. Wheeler, Judith Ann Dean, Federica Barzi

**Affiliations:** 1 Poche Centre for Indigenous Health, Faculty of Health, Medicine and Behavioural Sciences, The University of Queensland, Brisbane 4066, Australia; j.dean4@uq.edu.au (J.A.D.); f.barzi@uq.edu.au (F.B.); 2 School of Human Movement and Nutrition Sciences, Faculty of Health, Medicine and Behavioural Sciences, The University of Queensland, Brisbane 4072, Australia; kwheeler@uq.edu.au

**Keywords:** indigenous maternal health, perinatal health promotion, Aboriginal Community Controlled Health Organisations, SNAPS(o), maternal health equity, culturally safe care

## Abstract

**Highlights:**

**Public health relevance—How does this work relate to a public health issue?**

- Indigenous-led, community-controlled health services play a central role in delivering culturally grounded perinatal health promotion.
- This review maps perinatal health promotion across smoking, nutrition, alcohol, physical activity, and social and emotional wellbeing across Australia, Aotearoa New Zealand, Canada, and the United States.

**Public health significance—Why is this work of significance to public health?**

- The evidence base is dominated by tobacco-focused interventions, with limited evaluation of broader determinants of maternal and infant health.
- Indigenous community-controlled programs in Australia deliver holistic, multi-component models that are under-represented in the peer-reviewed literature.

**Public health implications—What are the key implications or messages for practitioners, policy makers and/or researchers in public health?**

- Evaluation frameworks must better reflect Indigenous models of care, including cultural safety, relational engagement, and integrated service delivery.
- Expanding evaluation beyond smoking to encompass holistic perinatal wellbeing is critical to informing equitable maternal health systems.

**Abstract:**

Indigenous women experience a disproportionate burden of adverse perinatal health outcomes, yet the extent and nature of health promotion interventions addressing modifiable behavioural and social determinants remain poorly synthesised. This scoping review mapped smoking, nutrition, alcohol, physical exercise, and social and emotional wellbeing (SNAPS(o))-related perinatal health promotion programs delivered through Australian Aboriginal Community Controlled Health Organisations (ACCHOs), supplemented by relevant peer-reviewed evidence identified across Australia, Aotearoa New Zealand, Canada, and the United States. A two-phase design looked at peer-reviewed literature from January 2010 to January 2025 across PubMed, CINAHL, and the Cochrane Library, followed by a structured review of Aboriginal Community Controlled Health Organisation (ACCHO) websites in Australia (*n* = 145). Data were extracted on program characteristics, SNAPS(o) components, implementation models, and evaluation outcomes. Findings were synthesised using content analysis. Thirty-four programs were identified in total, most delivered through ACCHOs (*n* = 26) and predominantly implemented in Australia (*n* = 29). Smoking was the most frequently addressed component (*n* = 18, 55%), while nutrition and social and emotional wellbeing were each included in 27% of programs (*n* = 9), physical exercise in 18% (*n* = 6), and alcohol in 15% (*n* = 5). Grey-literature programs more commonly reflected multi-component, holistic models compared with peer-reviewed studies and formal evaluations. Only 10 programs had identifiable formal evaluation evidence, including published or publicly reported evaluations, almost all of which were identified through academic sources. Evaluations focused primarily on tobacco-related behavioural outcomes, with limited reporting of sustained maternal or infant health endpoints. The perinatal SNAPS(o) intervention landscape for Indigenous women is characterised by strong community-controlled delivery but limited published evaluation, particularly of integrated models implemented within ACCHOs. The concentration of evidence on smoking cessation highlights a need to expand evaluation across broader domains of maternal wellbeing. Strengthening Indigenous-led evaluation frameworks and outcome measures that reflect holistic models of care is essential to advancing equitable and culturally grounded perinatal health systems.

## 1. Introduction

The perinatal period represents a critical window for maternal and infant health, globally. Despite advances in obstetric and neonatal care, preventable adverse maternal and infant health outcomes remain substantial worldwide [1]. A significant proportion of adverse outcomes are associated with modifiable behavioural, metabolic, and psychosocial risk factors, including, for instance, tobacco use, alcohol exposure, poor nutrition, low or high body mass index (BMI), and untreated mental health conditions [1,2,3,4]. Addressing these factors during pregnancy and the early postnatal period is widely recognised as an effective strategy to improve birth outcomes and reduce long-term disease burden [5,6].

The Developmental Origins of Health and Disease (DOHaD) framework provides a foundation for better understanding this life stage. DOHaD research demonstrates that exposures during pregnancy and early infancy, including nutrition, stress, and substance use, can alter gene expression, influence placental function, and shape foetal development, thereby affecting susceptibility to chronic disease across the life course [7,8,9,10]. These findings underscore the importance of preventing harmful exposures and strengthening protective, modifiable factors during the perinatal period to influence both immediate and intergenerational health trajectories [7,8,9,10].

Globally, Indigenous peoples experience disproportionately poorer maternal and infant outcomes compared to non-Indigenous populations within the same countries [8,11,12,13]. Colonisation, dispossession, cultural disruption, and structural racism have produced enduring inequities in housing, food security, education, income, and access to culturally safe health care. These social determinants shape exposure to modifiable perinatal risk factors and contribute to persistent disparities in birth outcomes [11,12,13].

In Australia, Aboriginal and Torres Strait Islander peoples continue to demonstrate strength, resilience, and cultural continuity, despite the profound and ongoing impacts of colonisation. For Aboriginal and Torres Strait Islander communities, health is understood holistically, encompassing physical wellbeing, cultural identity, kinship ties, connection to, and care for, Country, and participation in language and cultural practices [14]. This relational view of health contrasts with biomedical models and underpins the design of Aboriginal Community Controlled Health Organisations (ACCHOs), which deliver culturally safe, community-led, wraparound care [14].

Despite these strengths, Aboriginal and Torres Strait Islander women continue to experience inequities in exposure to modifiable perinatal risk factors. Higher rates of smoking during pregnancy, elevated BMI, pre-existing and gestational diabetes, food insecurity, and psychological distress have been consistently reported [5,15,16]. These health factors are intertwined with systemic barriers, including racism in mainstream services, fragmented maternity care pathways, and limited access to culturally safe services. Together, these structural determinants can exacerbate risk and undermine wellbeing during a life stage that is socially and culturally significant, when maternal identity is strengthened and kinship ties are reinforced [5,15,16].

National strategies such as the Close the Gap initiative have sought to redress persistent health inequities between Indigenous and non-Indigenous populations in Australia. For example, Target Two of the National Agreement focuses on ensuring Aboriginal and Torres Strait Islander children are born healthy and strong [17]. Over the past decade, progress has been made, including a reduction in infant mortality and increases in the proportion of babies born at a healthy birth weight [18]. These gains provide a foundation for future action, yet challenges remain. Rates of pre-term birth, low birth weight, and small-for-gestational-age infants continue to be higher among Aboriginal and Torres Strait Islander populations, underscoring the need for sustained and enhanced interventions. Strengthening culturally safe, community-led programs during the perinatal period is essential to accelerate this progress and close the gap in maternal and infant outcomes [18].

Within other high-income settler-colonial nations, particularly Aotearoa New Zealand, Canada, and the United States, similar colonial histories and health system structures have produced comparable patterns of inequity for Indigenous mothers and babies [11,12,13,19]. Focusing on these contexts enables meaningful comparison across countries shaped by parallel governance arrangements, Indigenous health policies, and community-controlled models of care, while maintaining relevance to the Australian setting.

Internationally, academic research in countries such as Aotearoa New Zealand [20], Canada [21], and the United States [22,23,24] has described perinatal interventions for Indigenous women, but the scope, delivery, and outcomes of these programs have not been systematically mapped alongside the program delivered in the Australian community-controlled context. This creates a fragmented evidence base and limits opportunities to translate community practice into academic and policy frameworks for Aboriginal and Torres Strait Islander women.

In this review, modifiable perinatal risk factors are framed using the acronym SNAPS(o): smoking, nutrition, alcohol, physical exercise, and social and emotional wellbeing. This approach builds on the work of Canuto et al. (2021) [25], who mapped SNAPS components within Australian health promotion programs [25]. However, the present review narrows the focus specifically to the perinatal period and to programs designed for Indigenous women within comparable settler-colonial contexts. The SNAPS(o) domains reflect both community priorities and national policy frameworks [14,17,26]. Despite the clear importance of these domains, there has been limited synthesis of how SNAPS(o)-related health promotion programs are implemented for Indigenous women during the perinatal period.

The limited peer-reviewed literature identified in the academic database search suggested that existing published evidence may not fully capture the breadth of community-led perinatal health promotion initiatives. Consequently, this review incorporated a grey-literature phase to map SNAPS(o)-related programs delivered through ACCHOs, enabling a more comprehensive understanding of intervention activity within community-controlled health services.

The objective of this review was to identify and describe the scope and focus of SNAPS(o) health promotion interventions designed for Indigenous women during the perinatal period, and to examine their implementation and reported maternal and infant outcomes across Australia, Aotearoa New Zealand, Canada, and the United States. Specifically, this review asked: what SNAPS(o)-related perinatal health promotion programs for Indigenous women have been described within international peer-reviewed literature and Australian community-controlled grey literature, and how have these programs been implemented and evaluated? This review looks at international peer-reviewed evidence with a focus on Australian ACCHO grey-literature analysis, to better understand how SNAPS(o)-related perinatal health promotion is implemented across academic, as well as community-controlled, settings. The review also identifies gaps in evaluation and reporting relevant to future research, policy, and program development.

## 2. Methods

### 2.1. Study Design

This scoping review followed the methodological framework developed by Arksey and O’Malley (2005) [27] and further refined by Levac, Colquhoun, and O’Brien (2010) [28]. It was conducted in accordance with the Preferred Reporting Items for Systematic Reviews and Meta-Analyses extension for Scoping Reviews (PRISMA-ScR) [19] and guided by the Joanna Briggs Institute (JBI) Manual for Evidence Synthesis. The PRISMA-ScR checklist can be found in File S1: PRISMA-ScR Checklist. This review also followed best-practice guidelines for applying systematic review methods to grey literature, outlined in the four-stage search strategies set out by Godin et al. (2015) [29].

The review sought evidence describing SNAPS(o)-related perinatal health promotion interventions delivered to Indigenous women across Australia, Aotearoa New Zealand, Canada, and the United States, including programs implemented through Aboriginal Community Controlled Health Organisations. Eligible sources included peer-reviewed publications, formal evaluation reports, and grey-literature documents reporting on one or more of the following: (1) program development, delivery, and implementation characteristics; (2) the specific SNAPS(o) components addressed; (3) feasibility, accessibility, uptake, or effectiveness, including reported maternal and infant outcomes and evaluation methods; and (4) identified gaps in implementation, reporting, or outcome measurement.

To distinguish between formally published academic evidence and community-controlled program documentation, the review was conducted in two distinct but complementary phases. Phase 1 comprised a systematic search of peer-reviewed databases to locate academic studies describing SNAPS(o)-related perinatal programs for Indigenous women in Australia, Aotearoa New Zealand, Canada, and the United States. These countries were selected due to their shared settler-colonial histories, comparable Indigenous governance structures, and similar health system contexts, enabling meaningful comparison across jurisdictions facing parallel structural determinants of maternal health inequities. Phase 2 involved a structured review of Aboriginal Community Controlled Health Organisation (ACCHO) websites across Australia, to identify grey literature documenting community-led health promotion programs. Integrating these phases enabled a triangulated analysis of both academic and community-sourced evidence, offering a more comprehensive understanding of the reach, design, and effectiveness of relevant interventions.

### 2.2. Search Strategy

#### 2.2.1. Phase 1: Peer-Reviewed Database Search

We initiated Phase 1 with a limited search of relevant articles in PubMed and the Cumulative Index to Nursing and Allied Health Literature (CINAHL), to identify similar reviews and refine key search terms. Medical Subject Headings (MeSH) and keywords identified during this preliminary search informed the development of the final search strategy (Appendix A). The comprehensive search was undertaken by C.C. from January 2025–May 2025 across three academic databases: PubMed, CINAHL, and the Cochrane Library. The complete database search strategies are provided in Appendix A. Reference lists of included studies were also manually screened to identify additional sources. Articles published in English between January 2010 and January 2025 were included, to capture contemporary health promotion programs and reflect current service delivery contexts.

#### 2.2.2. Phase 2: Grey-Literature Search (ACCHOs)

All registered Aboriginal Community Controlled Health Services/Organisations (ACCHS/ACCHO) listed by the National Aboriginal Community Controlled Health Organisation (NACCHO), the national peak body representing Aboriginal community-controlled health services in Australia, were systematically reviewed. The Phase 2 search was conducted between November 2024 and May 2025, by C.C. A total of 145 organisation websites (and most recent annual reports if they were available on the website) were examined to identify programs addressing one or more SNAPS(o) components: smoking, nutrition, alcohol, physical exercise, and social and emotional wellbeing, relevant to the perinatal period.

Phase 2 intentionally focused on Australian Aboriginal Community Controlled Health Organisations (ACCHOs), due to the existence of a nationally identifiable network coordinated through the National Aboriginal Community Controlled Health Organisation (NACCHO), and the central role of ACCHOs in delivering culturally safe, community-controlled maternal healthcare for Aboriginal and Torres Strait Islander peoples. Equivalent nationally coordinated and publicly searchable Indigenous community-controlled organisational structures were not consistently identifiable across the other included countries. Consequently, the inclusion of Australian ACCHO grey literature alongside international peer-reviewed literature introduced methodological asymmetry across countries, which has been acknowledged in the interpretation of findings.

### 2.3. Inclusion Criteria

The inclusion criteria for Phase 1 are listed in Table 1 and those for Phase 2 are listed in Table 2. Inclusion was determined using the Population–Concept–Context (PCC) framework, as recommended by the Joanna Briggs Institute (JBI) for scoping reviews. The population of interest comprised Indigenous women residing in Australia (Phases 1 and 2), Aotearoa New Zealand, Canada, or the United States (Phase 1), during the perinatal period, from conception through to 12 months postpartum. Studies were eligible for inclusion if this population was explicitly described or if disaggregated data were provided. No included studies involved mixed-population cohorts with separately reported Indigenous subgroup analyses.

### 2.4. Data Extraction

**Figure 1:**
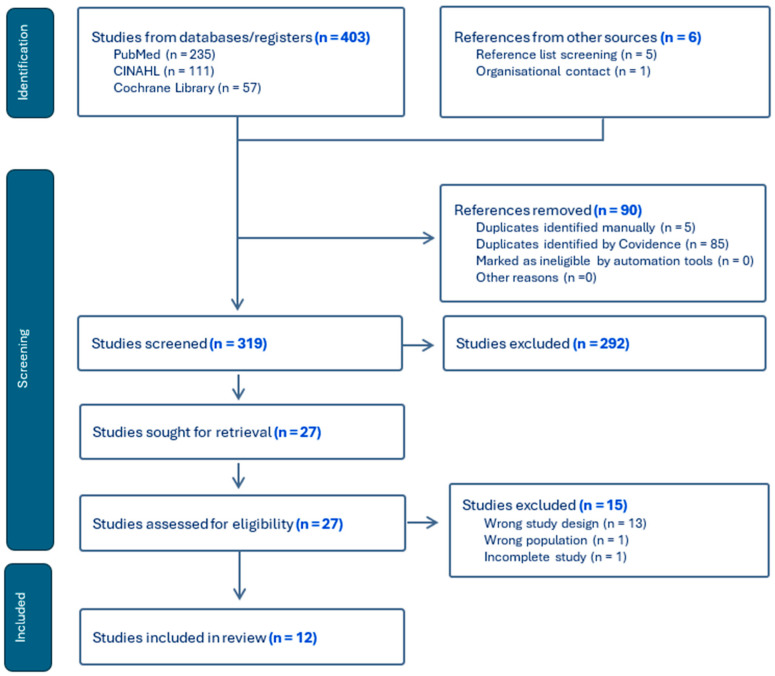
PRISMA Flow Chart.

A structured data-extraction form was developed and piloted, to ensure consistency across both phases. In Phase 1, database search results were imported into Covidence systematic review software (Veritas Health Innovation, Melbourne, VIC, Australia) for screening and de-duplication. Two reviewers (C.C. and K.W.) independently screened titles and abstracts, followed by full-text screening of potentially eligible articles. Discrepancies were resolved through discussion within the research team and consensus, and reasons for full-text exclusion were documented. The study selection process is illustrated in a PRISMA-ScR flow diagram (Figure 1).

In Phase 2, one reviewer (C.C.) independently screened and extracted program data from ACCHS websites and annual reports, using a standardised Excel template, with verification by three reviewers (KW, FB, and JD) to ensure accuracy. The reviewers met weekly to resolve discrepancies through discussion and consensus. This phase focused on program descriptions and implementation details, and a summary of program inclusion is presented in Table 3. All Phase 2 data were recorded in Excel and stored securely within institutional research data management systems. The Excel extraction sheet captured information on the program name and description, target population and perinatal stage, SNAPS(o) components addressed, delivery model and setting, evaluation status and outcomes (if available), and accessible resources such as annual reports, newsletters, or program evaluations. When insufficient information was available regarding specific programs identified online, organisations were contacted directly via phone call to request supplementary documentation. When insufficient information was available regarding specific programs identified online, organisations were contacted directly via phone call to request supplementary documentation. A total of four organisations were contacted to clarify program eligibility or obtain additional program information, of which three provided supplementary information. This step ensured data completeness and helped address gaps in the public reporting of community-driven programs.

The data-extraction tools for both phases were iteratively refined with input from the project team to incorporate emerging variables and ensure alignment with JBI guidelines. Across both phases, extracted data included bibliographic details (author, year, country, publication type); program characteristics (focus, components, duration, setting); delivery characteristics (provider type, Indigenous governance, community engagement); participant information (population, pregnancy stage, geographical classification); reported outcomes (maternal and infant health indicators, behavioural change, program engagement rates); and evaluation or monitoring methods.

### 2.5. Data Analysis and Integration

Across both phases, extracted data were mapped according to program type, SNAPS(o) components addressed, delivery characteristics, reported outcomes, and evaluation methods. Quantitative data were summarised descriptively. Program data from the peer-reviewed and grey-literature phases were merged to support integrated analysis across academic and community-controlled sources. Programs identified across both phases were cross-checked during extraction and synthesis to avoid duplicate inclusion, and no duplicate programs were identified across phases. Integrating findings across both phases also enabled comparison between peer-reviewed evidence and community-controlled program documentation, to identify areas of convergence, divergence, and under-representation within the published literature.

A coding framework aligned with the review questions was collaboratively developed by the research team, and applied using a structured Excel extraction template. Program data from both phases were manually coded across five domains: (1) program characteristics, including program focus, delivery approach, workforce, partnerships, and perinatal stage; (2) SNAPS(o) components addressed; (3) implementation characteristics, including accessibility, feasibility, acceptability, enablers, and barriers; (4) reported maternal, infant, and community outcomes; and (5) identified evidence gaps and limitations. Coding was initially undertaken by C.C. and iteratively refined through regular team discussions to support consistency and interpretation across both phases. Independent verification of coded data was undertaken by the broader research team during weekly review meetings, with discrepancies resolved through discussion and consensus. Contextual and relational content analysis approaches were applied [30,31] to examine patterns in program design, implementation approaches, evaluation practices, and reported outcomes. A thematic synthesis was subsequently undertaken to identify recurrent program features, common enablers and barriers, and areas of under-representation within the evidence base.

### 2.6. Methodological Quality Appraisal

Although quality appraisal is not mandatory in scoping reviews, the methodological quality of 10 was assessed to contextualise interpretation of findings. Two of the 12 eligible sources were study protocols; therefore, only 10 were eligible for appraisal. Appraisal was conducted using the Mixed Methods Appraisal Tool (MMAT) 2018 [32], which was selected due to the methodological heterogeneity of included qualitative, quantitative, and mixed-methods study designs. Two reviewers independently assessed study quality, with discrepancies resolved through discussion. Appraisal outcomes did not inform study inclusion or exclusion, but were used to interpret the strength, limitations, and consistency of reported outcomes. Methodological quality-appraisal findings are presented in Appendix B.

## 3. Results

### 3.1. Phase 1 Search Results

Database searches in Phase 1 identified 403 records, with an additional six records identified through other sources, including reference-list screening (*n* = 5) and organisational contact (*n* = 1). After removal of duplicates, 319 articles were screened by title and abstract. A total of 27 full-text articles were assessed for eligibility, of which 15 were excluded. Ten peer-reviewed studies and two study protocols met the inclusion criteria and were included in the review [2,20,22,23,24,33,34,35,36,37,38,39].

The additional study identified through organisational contact refers to a peer-reviewed publication identified during Phase 2 after ACCHOs were contacted to clarify whether any included programs had undergone formal evaluation or had associated published literature.

### 3.2. Phase 2 Search Results

The websites of all NACCHO-registered ACCHOs in Australia (as of March 2025) were screened, resulting in the identification of 135 potential programs for inclusion. After reviewing the program content, 22 programs were included in this review [40,41,42,43,44,45,46,47,48,49,50,51,52,53,54,55,56,57,58] (Table 3).

After screening and data extraction across both phases, all eligible sources were analysed collectively to enable integrated mapping of program characteristics, SNAPS(o) components, implementation approaches, and reported outcomes. A full list of ACCHO website mapping can be found in Supplementary Table S1. The sections that follow present synthesised findings across academic and grey-literature sources.

### 3.3. Overview of Program and Study Characteristics

A total of 34 eligible sources were included in the review, comprising 22 grey-literature program documents identified through Australian Aboriginal Community Controlled Health Organisation (ACCHO) websites and 12 peer-reviewed or formal evaluation publications describing perinatal health-promotion initiatives. The synthesis focused on the characteristics, implementation, and reported outcomes of the underlying programs described within these sources. Given the methodological asymmetry between the international peer-reviewed evidence base and the Australian ACCHO grey-literature dataset, findings are reported separately, where appropriate, to avoid overstating cross-country comparisons and to maintain contextual consistency in interpretation.

Most Australian programs described across both the peer-reviewed and grey-literature phases were delivered through ACCHOs or affiliated Aboriginal Community Controlled Health Services (*n* = 26), while the remainder were implemented through community outreach or non-ACCHO settings (*n* = 8). Australian programs and publications spanned the Australian Capital Territory (ACT), New South Wales (NSW), Northern Territory (NT), South Australia (SA), Western Australia (WA), Queensland (QLD), and Victoria (VIC). Within the peer-reviewed literature phase, four publications described programs implemented outside Australia, including three from the United States (including Alaska) [22,23,24] and one from Aotearoa New Zealand [20]. No eligible Canadian programs or publications were identified. The primary Indigenous populations represented included Aboriginal and Torres Strait Islander peoples in Australia, Māori in Aotearoa New Zealand, and Native American and Alaska Native communities in the United States.

Target participants varied across included sources. Most programs focused directly on pregnant women (*n* = 17), with an additional three programs explicitly targeting women during the broader perinatal period (*n* = 3). Five programs broadly targeted women (*n* = 5), while five adopted an entire community approach (*n* = 5). Programs classified as adopting an “entire community” approach were included only where program descriptions explicitly identified perinatal-focused components or activities targeting pregnancy and postpartum health outcomes, such as smoking reduction during pregnancy. Seven programs included health practitioners, either as the primary target or alongside women (*n* = 7). Four programs explicitly involved significant others or families (*n* = 4), and one program specifically targeted adolescent mothers (*n* = 1). Table 4 provides a summary overview of included source characteristics, including setting, target population, geographic location, and target Indigenous population, while detailed program-level characteristics are presented in Appendix C.

Methodological quality-appraisal findings for included peer-reviewed studies are summarised in Appendix B. Overall, study quality varied, with common methodological limitations including small sample sizes, attrition, limited follow-up periods, and challenges associated with sustaining participant engagement.

### 3.4. SNAPS(o) Components Addressed

Within the peer-reviewed literature, smoking cessation was the most frequently addressed SNAPS(o) component [2,20,22,23,33,35,36,37]. Most published interventions focused specifically on tobacco reduction during pregnancy among Aboriginal and Torres Strait Islander, Māori, Native American, and Alaska Native women [20,22,23,33,35,36,37]. Social- and emotional-wellbeing components were less commonly described within the peer-reviewed literature, and were generally embedded within broader maternal or family support initiatives [24,38]. Fewer peer-reviewed publications explicitly focused on nutrition [2,38], physical exercise [2], or alcohol use [2,38]. Several peer-reviewed programs addressed multiple SNAPS(o) components concurrently, particularly those embedded within maternal and child health or wraparound maternal-care models [24,38].

Within the Australian ACCHO grey literature, smoking cessation also represented the most frequently identified SNAPS(o) component, commonly delivered through national Tackling Indigenous Smoking (TIS) initiatives [39,40,43,47]. However, the grey literature additionally described a broader range of programs addressing nutrition [42,48,51,53,54,58], physical exercise [44,45,50,51,52], alcohol-related health promotion [42,49,53], and social and emotional wellbeing [44,45,46,50,52,56]. Several ACCHO programs incorporated multiple SNAPS(o) components within holistic maternal and child- health-service models [42,46,51,53]. Social and emotional wellbeing and physical exercise were the most frequently co-occurring SNAPS(o) components, identified together in six programs [38,44,45,50,51,52]. The combination of smoking, nutrition, and alcohol was identified in three Australian programs [38,42,53], while alcohol-related initiatives were almost always paired with smoking-related components [2,38,42,53].

When comparing Australian ACCHO grey-literature programs with Australian peer-reviewed publications only, a greater proportion of grey-literature programs addressed multiple SNAPS(o) components concurrently (8/22 programs), compared with Australian peer-reviewed publications (3/8 publications) [2,38,42,46,51,53]. Australian peer-reviewed publications more commonly focused on smoking-specific interventions [2,33,35,36,37], whereas ACCHO grey literature more frequently described holistic or integrated maternal-health promotion approaches delivered through community-controlled settings [42,46,51,53,54].

Overall, relatively few holistic, community-led Australian programs identified through the ACCHO grey literature were represented within the peer-reviewed evidence base. This pattern highlights ongoing gaps in the formal evaluation and publication of comprehensive SNAPS(o)-related programs delivered through Aboriginal Community Controlled Health Organisations. Table 5 provides an overview of the SNAPS(o) component distribution.

### 3.5. Focus and Implementation

Within the peer-reviewed literature, implementation approaches varied in structure, intensity, and delivery setting. Published interventions were primarily delivered through clinic-based maternal health services, structured home-visiting models, and community outreach programs. Smoking-cessation interventions represented the dominant implementation focus within the published literature [2,20,22,23,33,35,36,37], while several broader maternal and child health programs incorporated wraparound support approaches [24,34,38].

Structured home-visiting and outreach delivery models were prominent within the peer-reviewed evidence base [20,22,23,24,34]. These programs commonly described continuity of care commencing during pregnancy and extending into infancy or early childhood. The Family Spirit Program [24] and Healthy Pregnancies Project [23] both utilised structured outreach approaches, while Australian programs such as Baby One [34] and Strong Women, Strong Babies, Strong Culture [38] incorporated broader maternal-, family-, and community-support elements.

Several peer-reviewed publications highlighted culturally safe care, strengths-based approaches, and Indigenous workforce involvement [20,22,23,34,38]. However, implementation barriers and feasibility challenges were primarily reported within the peer-reviewed literature, including high loss to follow-up and limited intervention effect [23], low enrolment and feasibility concerns [22], and transportation and resource limitations [38].

Within the Australian ACCHO grey literature, clinic-based maternal and child health services represented the most commonly described delivery model. Programs such as the Women’s and Children’s Health Program [43] and Maternal and Child Health Unit [42] described multidisciplinary antenatal and postnatal care delivered through ACCHO clinical settings. These services commonly incorporated Aboriginal Health Workers, midwives, child-health nurses, and general practitioners, with several programs additionally describing shared-care arrangements with hospitals and referral pathways to allied health or specialist services [42,43,44,49,50,51,52,58].

Community-based group delivery was also commonly described within the ACCHO grey literature [41,44,50,51,52,56,57]. Physical exercise-focused initiatives were typically delivered through community gym or outdoor settings, and often incorporated informal yarning, peer support, and social engagement [44,45,50,51,52]. Nutrition-focused initiatives were similarly delivered through group-based settings incorporating cooking, education, or community-engagement activities [40,46,53,54,55,57,58]. Several ACCHO programs additionally described wraparound maternal and family support models integrating social and emotional wellbeing, maternal outreach, and continuity of care [44,50,51,52].

Most ACCHO grey-literature sources provided descriptive program information but limited formal evaluation detail. While accessibility strategies such as transport assistance, outreach, and incentive-based engagement were described [45,46,53], implementation barriers and feasibility challenges were rarely reported within publicly available program documents.

Compared with Australian peer-reviewed publications, Australian ACCHO grey literature more frequently described holistic, community-led maternal health-promotion models incorporating multidisciplinary care, outreach delivery, group-based activities, and integrated social- and emotional- wellbeing support [42,44,46,50,51,52,53]. In contrast, Australian peer-reviewed publications more commonly focused on individual intervention components, particularly smoking-cessation initiatives [2,33,35,36,37]. Overall, relatively few community-controlled models identified within the ACCHO grey literature appeared within the formal peer-reviewed evidence base.

### 3.6. Evaluation Design and Methodological Characteristics

Within the peer-reviewed literature, evaluation designs varied considerably in methodological approach and rigour. Quantitative randomised controlled trials (RCTs) were the most common formal evaluation design (*n* = 4), primarily assessing smoking-cessation interventions delivered through ACCHO settings, community outreach programs, or structured maternal-support models [22,23,24,35]. These studies typically employed individual or cluster randomisation, with smoking outcomes assessed using self-reported behavioural measures and, in some cases, biochemical validation [22,23,35]. Several studies reported methodological challenges including differential attrition, small sample sizes, incomplete follow-up, and limited statistical power to detect sustained behavioural change [22,23,35].

Two studies employed mixed-methods cluster or step-wedge designs integrating quantitative outcome measures with qualitative assessments of feasibility, acceptability, and implementation processes [33,37]. These studies triangulated behavioural outcomes with contextual implementation data, including workforce engagement, service integration, and practice change within community-controlled environments [33,37].

Three peer-reviewed studies utilised qualitative methodologies focusing on implementation fidelity, workforce capacity, cultural integration, governance structures, and participant experience [34,37,38]. These evaluations did not primarily assess behavioural endpoints, but provided detailed insight into enablers and barriers to program delivery, particularly within remote and community-based contexts [34,38].

In terms of delivery setting, peer-reviewed evaluations were conducted across both ACCHO clinical services and community outreach models [20,22,23,24,33,34,35,37,38]. Programs evaluated within ACCHO settings more commonly focused on workforce training, smoking-cessation support, clinical practice change, and integration into routine antenatal care [33,35,37], whereas outreach models frequently relied on home-visiting approaches, paraprofessional delivery, peer support, and relationship-based engagement strategies [20,23,24,34,38].

Within the Australian ACCHO grey literature, formal evaluation reporting was limited. Of the 22 ACCHO programs identified in Phase 2, only one program described a structured formal evaluation process through a published mixed-methods program evaluation [39]. This evaluation examined system-level outputs including program reach, exposure, referral pathways, workforce capacity, smoke-free policy adoption, and stakeholder perspectives [39].

Most ACCHO grey-literature sources focused primarily on program descriptions, service delivery models, and community engagement activities, rather than formal outcome evaluation. Although many programs described holistic maternal and family support approaches, detailed reporting of implementation processes, evaluation methods, participant outcomes, or long-term effectiveness was generally limited within publicly available program documentation.

Compared with Australian ACCHO grey literature, Australian peer-reviewed publications provided substantially greater methodological detail regarding study design, implementation processes, participant outcomes, and evaluation approaches [2,33,34,35,36,37,38]. In contrast, ACCHO grey literature more commonly documented service delivery activities, culturally safe care models, and community-led maternal health initiatives without accompanying formal evaluation data. This pattern highlights a substantial gap between the breadth of maternal health-promotion activity occurring within community-controlled settings and the availability of formally published evaluation evidence.

### 3.7. Reported Maternal, Infant and Behavioural Outcomes

Across the 10 formally evaluated programs identified in this review [20,22,23,24,33,34,35,38,39], outcomes varied considerably in scope, measurement, and reported effectiveness. The majority of evaluations focused on behavioural outcomes related to tobacco use during pregnancy (*n* = 7) [2,20,22,23,33,34,35,37,38], reflecting the dominance of smoking cessation within the SNAPS(o) evidence base. Fewer studies assessed broader maternal psychosocial outcomes (*n* = 2) [24,33], child developmental outcomes (*n* = 1) [24], or system-level knowledge and policy indicators (*n* = 1) [39]. Direct infant health outcomes, such as birthweight or gestational age, were infrequently reported as primary endpoints [20].

Among tobacco-focused interventions, findings were mixed. Several studies reported improvements in quit attempts or reductions in cigarette consumption during pregnancy [20,23,33]. For example, Gould et al. (2022) reported that 36% of pregnant women attempted quitting, with two women remaining smoke-free postpartum, alongside reductions in tobacco use among significant others [33]. Similarly, Glover et al. (2016) reported that 33% of participants quit smoking during pregnancy and 57% reduced consumption [20]. However, biochemically validated abstinence rates were generally modest, and some trials reported no statistically significant difference in cessation rates between intervention and usual care groups at follow-up [22,35]. While some studies observed increased quit attempts postpartum [23], sustained abstinence remained limited across several RCTs [22,23,35].

Mixed-methods evaluations conducted within ACCHO settings provided additional contextual insight [33,37]. Gould et al. (2022) reported a 13.6% validated abstinence rate at 12 weeks, alongside high acceptability and feasibility within participating ACCHOs and reported changes in routine clinical practice [33]. Qualitative findings across mixed-methods studies highlighted improvements in provider confidence, enhanced cultural safety, and increased integration of smoking-cessation support into antenatal care, even when behavioural endpoints were moderate [33,37].

Beyond tobacco use, broader maternal- and child-wellbeing outcomes were less frequently assessed, but demonstrated notable impacts when evaluated. Barlow et al. (2015) reported improvements in maternal parenting knowledge, increased locus of control, reduced depressive symptoms, and reductions in child behavioural problems sustained up to 36 months in a home-visiting program [24].

Qualitative evaluations [34,38] did not measure behavioural endpoints directly, but identified improvements in service engagement, strengthened relationships between Indigenous health workers and families, increased integration of cultural knowledge, and enhanced workforce capacity. These studies underscored the importance of relational delivery models, governance structures, and Indigenous leadership in shaping program effectiveness [34,38]. At a system level, the large-scale evaluation of the Tackling Indigenous Smoking program [39] demonstrated that more than 75% of reported activities increased knowledge of smoking harms, although behavioural change and smoke-free policy adoption varied across regions.

Overall, while several programs demonstrated promising improvements in knowledge, quit attempts, parenting capacity, and psychosocial wellbeing [20,24,33,37], robust evidence of sustained behavioural change and measurable infant-health outcomes was limited [22,23,35]. The concentration of evaluation within tobacco-focused interventions further suggests that other SNAPS(o) domains remain comparatively under-evaluated in relation to maternal and infant health outcomes. Table 6 outlines the evaluation designs, settings, outcomes assessed, and key findings across the formally evaluated programs included in this review.

### 3.8. Methodological Quality Appraisal Findings

Methodological quality appraisal of the 10 peer-reviewed studies identified variation in study quality across designs (Appendix B). Qualitative studies generally demonstrated strong methodological coherence and clearly described analytic processes [34,38], while several quantitative and mixed-methods studies reported limitations relating to attrition, lack of blinding, baseline group differences, and incomplete follow-up data [23,24,35]. Many studies focused primarily on feasibility, implementation, or acceptability outcomes, rather than long-term effectiveness measures [20,37,39]. Overall, the appraisal findings were used to contextualise interpretation of the evidence base, rather than determine study inclusion.

## 4. Discussion

This scoping review mapped 34 SNAPS(o)-aligned perinatal initiatives for Indigenous women across Australia, Aotearoa New Zealand, Canada, and the United States, with most programs delivered through ACCHOs yet only a minority supported by formal evaluation evidence. First, the published and publicly available intervention landscape was dominated by tobacco-related initiatives, while nutrition, alcohol, physical exercise, and social and emotional wellbeing were less frequently and less consistently targeted. Second, grey literature more commonly reflected integrated, community-led SNAPS(o) models than the peer-reviewed evidence base, indicating a disconnect between what is delivered in practice and what is formally evaluated and published. Third, where evaluation evidence existed, it concentrated on smoking-cessation outcomes and frequently reported feasibility and implementation barriers that likely constrain effect sizes and sustained behavioural change, particularly in trials relying on biomedical endpoints and longer-term follow-up [20,22,23,24,33,34,35,38,39].

This review highlights a persistent imbalance in the perinatal health-promotion evidence base for Indigenous women, with smoking cessation representing the dominant focus relative to other modifiable risk factors. The concentration of evaluated interventions on tobacco likely reflects the historical prioritisation of smoking reduction within maternal and child health policy, the availability of dedicated smoking-cessation funding streams, and the relative ease of measuring smoking-related behavioural outcomes within conventional evaluation frameworks. In contrast, alcohol-related interventions were far less frequently identified, which may reflect the tendency for alcohol support to be embedded within broader holistic or social and emotional wellbeing programs, rather than delivered or evaluated as standalone interventions. This pattern may reflect both genuine differences in service delivery models, with ACCHOs more commonly implementing holistic and integrated approaches to care, and publication or evaluation biases whereby single-component interventions are more readily evaluated, standardised, and published within conventional academic research frameworks.

Despite the known inter-relationship between behavioural risks and social and emotional wellbeing during the perinatal period [7,8,9,10], the broader SNAPS(o) framework does not appear to be consistently operationalised across program design or evaluation. Consequently, the evidence base remains characterised by a narrow evaluative focus on smoking outcomes over multidomain maternal wellbeing and infant health outcomes, potentially limiting the capacity of research to inform integrated, culturally grounded perinatal care models [20,22,23,24,33,34,35,38,39].

A key contribution of this review is the triangulation of academic studies with ACCHO-identified programs, which brings practice-based models into clearer view. The grey literature demonstrated a strong emphasis on holistic delivery, frequently combining social- and emotional-wellbeing supports with group-based physical exercise, nutrition programs, or wraparound maternal and child health services [40,41,42,43,44,45,46,47,48,49,50,51,52,53,54,55,56,57,58]. In contrast, peer-reviewed studies more often examined single-component interventions, particularly smoking cessation [2,20,22,23,24,33,34,35,36,37,38,39,59,60]. As a result, the peer-reviewed evidence base may systematically under-represent community-controlled, integrated programs that are likely to be more aligned with Indigenous concepts of health, relational care, and cultural safety [61].

It is also important to recognise that some broader Indigenous-led maternity and continuity-of-care programs may not have been captured where SNAPS(o)-related intervention components or outcomes were not explicitly described within publications or program documentation. For example, Kildea et al. (2019) [5] reported maternal and infant outcomes associated with the Birthing in Our Community (BiOC) program; however, the publication did not explicitly evaluate, or report intervention outcomes aligned with the predefined SNAPS(o) eligibility domains applied in this review. While such programs may incorporate holistic health-promotion activities relevant to smoking, nutrition, physical activity, and social and emotional wellbeing, these components may not always be reported within discrete behavioural-intervention frameworks. This highlights both the strengths and limitations of using predefined behavioural eligibility criteria and reinforces the importance of interpreting findings within the broader context of Indigenous community-controlled maternity care.

Across included sources, three implementation models were evident: clinic-based multidisciplinary maternal and child health care, structured home-visiting and outreach models, and community-based group programs. The clinic-based ACCHO models described the integration of midwifery, Aboriginal Health Workers, child-health nursing, GPs, and referral pathways, often with shared-care arrangements [44,50,52]. These models appear well positioned to embed SNAPS(o) support into routine antenatal and postnatal care. Their likely mechanisms of action include continuity, trust, and timely escalation to allied health and specialist services.

Home-visiting and outreach models were prominent across structured maternal and early childhood programs, including those commencing in pregnancy and continuing through infancy or early childhood [20,22,34,40]. The evidence suggests these models are frequently justified with respect to relational engagement and sustained support, which are consistent with Indigenous models of care prioritising family-centred practice and culturally safe relationships. Notably, the strongest signals for broader psychosocial and parenting outcomes in this dataset were observed in a home-visiting program evaluated using a rigorous RCT design, with effects sustained beyond infancy [24].

Community-based group programs also featured prominently, including yarning-based maternal groups, movement programs, and nutrition skill-building initiatives [38,44,45,50,51,52,54,58]. These models likely operate through social connection, peer support, and reduced isolation, which are plausible pathways to improved social and emotional wellbeing and sustained engagement [4]. The frequent co-occurrence of social and emotional wellbeing with physical exercise in this review supports this interpretation, and suggests an opportunity to frame physical exercise programs as mental health- and social-wellbeing interventions that can also influence metabolic and behavioural risk [38,44,45,50,51,52]. Yet, this pathway remains under-evaluated in the peer-reviewed literature, limiting the ability to quantify or compare impacts across settings.

Only 10 of the 34 included programs or studies had formal evaluation evidence, and just one evaluated program was identified through ACCHO website screening [20,22,23,24,33,34,35,38,39]. This gap reflects structural constraints in evaluation funding, workforce capacity, data infrastructure, and the fit of conventional research designs within community-controlled service delivery contexts. Several RCTs and feasibility trials reported challenges including small samples, attrition, differential follow-up, and limited power to detect sustained changes [22,23,35]. These limitations may both reduce the ability of studies to detect meaningful program effects and reflect genuine implementation challenges associated with sustaining engagement and delivering interventions within real-world service settings. Nevertheless, improvements in areas such as engagement, knowledge, confidence, and service integration were frequently reported in mixed-methods and qualitative evaluations, even when measurable behavioural outcomes were modest [33,34,37,38,39].

The disconnect observed between Indigenous community-controlled program delivery and formal academic evaluation is likely shaped by multiple intersecting structural and methodological factors. Community-controlled services frequently operate within constrained funding environments where service delivery priorities understandably take precedence over resource-intensive research and evaluation activities. Short-term funding cycles, workforce pressures, and limited access to dedicated evaluation infrastructure may further restrict opportunities for formal program evaluation and publication. In addition, conventional biomedical evaluation frameworks may not align well with Indigenous models of care that prioritise relational engagement, cultural safety, community trust, and holistic wellbeing outcomes that are not easily captured through standard quantitative measures. Differences between academic research priorities and community-defined indicators of success may also influence which programs are formally evaluated and published within peer-reviewed literature.

While the current review did not formally examine predictors of evaluation, programs with published evaluation evidence appeared more commonly associated with academic partnerships, externally funded research initiatives, or tobacco-focused intervention streams. However, available academic and grey literature frequently lacked sufficient detail regarding organisational capacity, workforce resourcing, funding structures, program maturity, or evaluation infrastructure to support systematic comparison between evaluated and non-evaluated programs. This highlights an important area for future research examining the structural and institutional factors that shape which Indigenous-led perinatal programs undergo formal evaluation and publication.

The field would benefit from an expanded definition of “effectiveness” that aligns with Indigenous priorities and program logic. Evidence in this review suggests that acceptability, cultural safety, relationship quality, workforce confidence, and integration into routine care are critical proximal outcomes that may precede behavioural change, and may represent important endpoints in their own right [33,34,37,38,39]. These outcomes may also function as mechanisms supporting sustained engagement with maternity services, earlier identification of health concerns, continuity of care, and ongoing participation in health-promoting behaviours during the perinatal period. Within Indigenous models of care, culturally appropriate outcome measures may also include women’s experiences of trust and relational safety, engagement with services, connection to family and community supports, self-determined goal attainment, cultural connectedness, and perceived empowerment throughout pregnancy and the postpartum period. Future investment should support community-controlled organisations to lead or co-design evaluation activities, with dedicated resourcing for culturally grounded monitoring, evaluation, and knowledge translation embedded within program funding models.

## 5. Limitations

Despite the breadth of the search, some relevant programs may have been missed due to inconsistent online reporting, limited web presence, or restricted access to internal documentation. Although no eligible Canadian programs were identified within the current review, this finding should be interpreted cautiously. The absence of identified programs may reflect limitations associated with database indexing, publication pathways, and the under-representation of Indigenous community-led initiatives within peer-reviewed literature, rather than a true absence of perinatal health-promotion activity within Canadian Indigenous health contexts. Community-based programs operating outside conventional academic-publication pathways may therefore not have been captured within the current search framework. Moreover, heterogeneity in program descriptions and evaluation methods may limit comparability across studies.

In addition, this review was designed as a descriptive mapping exercise, and did not include formal comparative analysis of organisational or structural factors associated with program evaluation. Publicly available program documentation frequently lacked sufficient detail regarding funding structures, organisational capacity, workforce resourcing, program maturity, or evaluation infrastructure to support systematic comparison between evaluated and non-evaluated programs. These challenges were addressed by drawing from multiple data sources, contacting organisations directly to supplement any missing information, and applying inclusive eligibility criteria to capture a broad range of intervention types. The grey-literature search was restricted to ACCHOs in Australia only. Grey-literature sources from Aotearoa New Zealand, Canada, and the United States were not systematically searched, which may have resulted in an under-representation of community-led programs in these settings.

## 6. Conclusions

The current SNAPS(o) perinatal intervention landscape for Indigenous women is characterised by strong community-controlled delivery capacity, especially through ACCHOs, but a comparatively thin peer-reviewed evaluation base. Tobacco-focused interventions dominate evaluation evidence, while integrated SNAPS(o) models addressing social and emotional wellbeing, nutrition, physical exercise, and alcohol remain under-represented in published research, despite their presence in practice. Advancing the field will require evaluation approaches that match community-led program logic, strengthen reporting of holistic outcomes, and expand publication of ACCHO-delivered models to better inform equitable, culturally safe maternal-health systems.

## Figures and Tables

**Table 1:** Inclusion and exclusion criteria for Phase 1 (academic database).

Criteria	Include	Exclude
Date	Studies published between 2010 and 2025	Studies published before 2010
Intervention	Studies where at least one SNAPS(o)-related intervention component was explicitly described within the publication or program documentation; Relevant to the perinatal context (pregnancy to 12 months postpartum)	Studies not related to at least one SNAPS(o) element; Studies not relevant to the perinatal context
Location	Australia, Canada, Aotearoa New Zealand, United States	Other countries
Population	Studies involving Indigenous women during the perinatal period, or studies reporting findings specific to Indigenous women through disaggregated analyses	Studies not reporting findings specific to Indigenous women
	Women in the perinatal period (pregnancy to 12 months postpartum)	Women not in the perinatal period
Publication	Peer reviewed journal articles; Primary empirical qualitative and quantitative studies	Grey literature, reports, conference proceedings, scoping reviews, systematic reviews, literature reviews or any other syntheses
Publication language	English	Non-English

**Table 2:** Inclusion and exclusion criteria for Phase 2 (ACCHO websites).

Criteria	Include	Exclude
Intervention	Studies related to at least one SNAPS(o) element; Relevant to the perinatal context (pregnancy to 12 months postpartum)	Studies not related to at least one SNAPS(o) element; Studies not relevant to the perinatal context
Location	Australia	Other countries
Population	Aboriginal and Torres Strait Islander women	Non-Indigenous women
	Women in the perinatal period (pregnancy to 12 months postpartum)	Women not in the perinatal period
Publication	Grey literature, reports, annual reports, internal or external evaluation documents, program flyers, peer reviewed papers	N/A
Language	English	Non-English

**Table 3:** Phase 2 (ACCHO website) program selection summary.

Phase 2 Screening Summary	Total
ACCHO/ACCHs websites screened	145
Programs identified for screening	135
Excluded: umbrella organisations/peak bodies representing ACCHOs already included elsewhere in the search	8
Excluded: no identifiable SNAPS(o)-related component specific to the perinatal period	105
Programs included in review	22

**Table 4:** Overview Characteristics.

Characteristic	*n* (%)
ACCHO grey literature	22 (64.7%)
Peer-reviewed/formal evaluations	12 (35.3%)
Setting	
ACCHO/ACCHs	26 (76.5%)
Community outreach	8 (23.5)
Target participants	
Pregnant women	17 (50.00%)
Women in perinatal period	3 (8.8%)
Women broadly	5 (14.7%)
Entire community	5 (14.7%)
Health practitioners	7 (20.6%)
Significant others/families	4 (11.8%)
Adolescent mothers	1 (2.9%)
Country	
Australia	29 (85.3%)
United States (US)	4 (11.8%)
Aotearoa New Zealand	1 (2.9%)

**Table 5:** SNAPS(o) Components.

Phase	Reference	Program Name/Intervention	S	N	A	P	S(o)
Phase 2	Winnunga Nimmityjah Aboriginal Health and Community Services [54]	Womens Group				✔	✔
Phase 2	Winnunga Nimmityjah Aboriginal Health and Community Services [54]	Healthy Cooking Group		✔			
Phase 2	Winnunga Nimmityjah Aboriginal Health and Community Services [54]	Pregnancy Group	✔	✔	✔		
Phase 2	Armajun Aboriginal Health Service [55]	Armidale Mums and Bubs		✔			✔
Phase 2	Biripi Aboriginal Corporation [41]	Child and Family Health Program	✔				
Phase 2	Bullinah Aboriginal Health Service [56]	Mums and Bubs Yarn Up Program					✔
Phase 2	Durri Aboriginal Corporation Medical Service [57]	Aboriginal Maternal and Infant Health Services		✔			
Phase 2	Wurli Wurlinjang Aboriginal Corporation [43]	Tackling Indigenous Smoking (TIS) Program	✔				
Phase 2	Nganampa Health Council Incorporated [47]	Tackling Indigenous Smoking (TIS) Program—Tjikita—Nyuntu Ngayuku Malpa Wiya	✔				
Phase 2	Tullawon Health Service Incorporated [48]	Child and Maternal Health Program		✔			
Phase 2	Beagle Bay Community Incorporated (KAMS Clinic) [40]	Australian Family Partnership Program					✔
Phase 2	Beagle Bay Community Incorporated (KAMS Clinic) [40]	Tackling Indigenous Smoking (TIS) Program	✔				
Phase 2	Derbarl Yerrigan Health Service Aboriginal Corporation [42]	Maternal and Child Health Program	✔	✔	✔		
Phase 2	Ngangganawili Aboriginal Community Controlled Health & Medical [53]	Maternal and Child Health Program	✔	✔	✔		
Phase 2	Nindilingarri Cultural Health Service Incorporated [46]	Mums, Bubs and Strong Families Health Promotion Program	✔		✔		
Phase 2	Ord Valley Aboriginal Health Services Aboriginal Corporation (member of KAMS) [49]	The Fetal Alcohol Spectrum Disorder (FASD) team			✔		
Phase 2	Kirrae Health Services Incorporated [45]	Deadly Walkers				✔	
Phase 2	Aboriginal and Torres Strait Islander Community Health Service Brisbane Limited [50]	Deadly Fit Mums				✔	
Phase 2	Carbal Aboriginal and Torres Strait Islander Health Services Ltd. [51]	Strong born Mums group		✔			✔
Phase 2	Institute for Urban Indigenous Health Ltd. [52]	Deadly Fit Mums				✔	
Phase 2	Townsville Aboriginal and Torres Strait Islander Corporation for Health Services [58]	The Maternal and Child Health team		✔			
Phase 2	Yulu-Burri-Ba Aboriginal Corporation for Community Health [44]	Deadly Fit Mums				✔	
Phase 1	Gould et al. (2022) [33]	SISTAQUIT	✔				
Phase 1	Askew et al. (2019) [2]	Smoking-cessation health promotion intervention	✔				✔
Phase 1	Barlow et al. (2015) [24]	Family Spirit Program	✔				✔
Phase 1	Campbell et al. (2018) [34]	Baby One Program					✔
Phase 1	Eades et al. (2013) [35]	Smoking-cessation health promotion intervention	✔				
Phase 1	Patten et al. (2020) [23]	Healthy Pregnancies	✔				
Phase 1	Bar-Zeev et al. (2017) [36]	ICANQUIT (Protocol)	✔				
Phase 1	Gould et al. (2019) [37]	ICANQUIT (Full study)	✔				
Phase 1	Glover et al. (2016) [20]	“Aunties” cessation program	✔				
Phase 1	Patten et al. (2010) [22]	Smoking-cessation health promotion intervention	✔				
Phase 1	Lowell et al. (2015) [38]	Strong women, strong babies, strong culture program	✔			✔	✔
Phase 1	Culturally Inclusive Research Centre Australia (2025) [39]	Evaluation of the Tackling Indigenous Smoking Program 2023–24 to 2026–27	✔				

**Table 6:** Outcomes, feasibility.

Author (Year)	Setting	Study Design	Evaluation Approach	Outcomes Assessed	Key Results
Gould et al. (2022) [33]	ACCHO/ACCHs	Mixed methods	Cluster randomised controlled trial with embedded mixed-methods evaluation. Quantitative outcomes (smoking reduction, depression scores) were triangulated with qualitative interviews assessing feasibility, acceptability, and participant experience.	Smoking behaviour	36% of pregnant women attempted quitting; two remained smoke-free postpartum; reductions in tobacco use reported, particularly among significant others.
Barlow et al. (2015) [24]	Community Outreach	Quantitative RCT	Stratified block randomised controlled trial (1:1 allocation). Independent blinded assessors conducted observational assessments; retention and lesson completion rates were reported.	Maternal and child wellbeing	Improved parenting knowledge and locus of control; reduced depressive symptoms and child behavioural problems sustained up to 36 months.
Campbell et al. (2018) [34]	Community Outreach	Qualitative	Qualitative evaluation using semi-structured interviews and focus groups. Grounded Theory–informed data collection; thematic analysis conducted using NVivo.	Program implementation	High uptake across nine remote communities; effectiveness linked to Indigenous Health worker–family relationships, yarning, workforce strengthening.
Eades et al. (2013) [35]	ACCHO/ACCHs	Quantitative RCT	Randomised controlled trial with week-based allocation. Smoking-related behavioural outcomes assessed; follow-up and retention rates reported.	Smoking cessation	No significant difference in smoking rates at 36 weeks between intervention and usual care.
Patten et al. (2020) [23]	Community Outreach	Quantitative RCT	Cluster randomised controlled trial with computer-generated allocation. Adjusted analysis for baseline differences; assessed tobacco use outcomes and participation rates.	Tobacco use	High program reach (73%); no reduction in tobacco use, but increased quit attempts postpartum.
Gould et al. (2019) [37]	ACCHO/ACCHs	Mixed methods	Mixed-methods step-wedge cluster feasibility trial. Quantitative feasibility outcomes triangulated with qualitative interviews, interpreted using COM-B and TDF frameworks.	Smoking abstinence, feasibility	13.6% validated abstinence at 12 weeks; intervention acceptable and feasible within ACCHSs; changes in routine practice reported.
Glover et al. (2016) [20]	Community Outreach	Quantitative descriptive	Quantitative descriptive feasibility study without control group. Self-reported smoking measures and medical record–derived birth outcomes assessed.	Smoking behaviour	33% quit smoking during pregnancy; 57% reduced consumption; increased use of cessation supports.
Patten et al. (2010) [22]	Community Outreach	Quantitative RCT	Randomised controlled trial assessing feasibility of cessation intervention; tobacco outcomes measured, with intervention and assessment delivered by same counsellor.	Feasibility, acceptability, smoking abstinence	Very low participation (12% of eligible women, 35/293). Retention was high in intervention (71%) and control (94%). Biochemically confirmed abstinence at follow-up was 0% (intervention) and 6% (control).
Lowell et al. (2015) [38]	Community Outreach	Qualitative	Qualitative evaluation using semi-structured interviews and document analysis. Inductive coding and thematic analysis with iterative researcher verification.	Cultural practice integration	Variable inclusion of Aboriginal knowledge across sites; governance, partnerships, and resourcing influenced implementation.
CIRCA (2025) [39]	ACCHO/ACCHs	Mixed methods	Mixed-methods program evaluation. Quantitative program output indicators (reach, referrals, staffing levels) analysed alongside qualitative interviews and focus groups examining barriers, enablers, and cultural safety.	Knowledge, attitudes, policy outcomes	>75% of activities increased knowledge of smoking harms; variable success in behaviour changes and smoke-free policy adoption across regions.

## Data Availability

All data generated or analysed during this study are included in this published article and its Supplementary Information Files.

## Supplementary Materials

The following supporting information can be downloaded at: https://www.mdpi.com/article/10.3390/ijerph23060746/s1. Table S1: Phase 2 Search, File S1: PRISMA-ScR Checklist.

## Appendix A. Search Terms

**Database**	**Terms**	**Date Searched**
CINAHL	(((MH “australian aboriginal and torres strait islander peoples+”) OR “torres strait islander” OR (MH “american indian or alaska native+”) OR “native american” OR (MH “native hawaiian or pacific islander+”) OR ((MH inuit+) OR inuit OR inuits) OR metis OR “alaska native*” OR (MH oceanians+) OR (MH “maori people+”) OR maori*) AND ((((MH pregnancy+) OR pregnan* OR (MH “pregnant people+”) OR prenatal OR perinatal OR postnatal OR (MH “prenatal care+”)) AND (MH “perinatal care+”)) OR (MH “postnatal care+”) OR (antenatal OR antenatally) OR (MH “postpartum period+”) OR ((MH “postpartum period+”) OR (postpartum AND period) OR “postpartum period” OR postpartum) OR (MH parturition+) OR childbirth OR birthing)) AND ((MH exercise+) OR exercise OR exercises OR (MH “exercise therapy+”) OR (exercise OR therapy) OR “exercise therapy” OR exercising OR “exercise s” OR exercised OR exerciser OR exercisers OR “aerobic exercise” OR ((MH “motor activity+”) OR (motor AND activity) OR “motor activity”) OR “physical activity” OR (MH “health promotion+”) OR (health AND promotion) OR “health promotion” OR (MH “smoking cessation+”) OR ((MH “tobacco use+”) OR tobacco OR “tobacco use”) OR nutrition OR (MH “nutritional status+”) OR (MH “diet, food, and nutrition+”) OR (MH “alcohol drinking+”) OR (MH “social support+”) OR well-being OR “mental health” OR “social health” OR (MH “mental health+”))	January 2025–May 2025
Cochrane	((([mh “australian aboriginal and torres strait islander peoples”] OR “torres strait islander” OR [mh “american indian or alaska native”] OR (“native” NEXT american*) OR [mh “native hawaiian or pacific islander”] OR ([mh inuit] OR inuit OR inuits) OR metis OR (“alaska” NEXT native*) OR [mh oceanians] OR [mh “maori people”] OR maori*) AND ((([mh pregnancy] OR pregnan* OR [mh “pregnant people”] OR prenatal OR perinatal OR postnatal OR [mh “prenatal care”]) AND [mh “perinatal care”]) OR [mh “postnatal care”] OR (antenatal OR antenatally) OR [mh “postpartum period”] OR ([mh “postpartum period”] OR (postpartum AND period) OR “postpartum period” OR postpartum) OR [mh parturition] OR childbirth OR birthing)) AND ([mh exercise] OR exercise OR exercises OR [mh “exercise therapy”] OR (exercise OR therapy) OR “exercise therapy” OR exercising OR “exercise s” OR exercised OR exerciser OR exercisers OR “aerobic exercise” OR ([mh “motor activity”] OR (motor AND activity) OR “motor activity”) OR “physical activity” OR [mh “health promotion”] OR (health AND promotion) OR “health promotion” OR [mh “smoking cessation”] OR ([mh “tobacco use”] OR tobacco OR “tobacco use”) OR nutrition OR [mh “nutritional status”] OR [mh “diet, food, and nutrition”] OR [mh “alcohol drinking”] OR [mh “social support”] OR well-being OR “mental health” OR “social health” OR [mh “mental health”])):ti,ab,kw” (Word variations have been searched)	January 2025–May 2025
PubMed	((“australian aboriginal and torres strait islander peoples”[MeSH Terms] OR “torres strait islander”[All Fields] OR “american indian or alaska native”[MeSH Terms] OR “native american*”[All Fields] OR “native hawaiian or pacific islander”[MeSH Terms] OR (“inuit”[MeSH Terms] OR “inuit”[All Fields] OR “inuits”[All Fields]) OR “metis”[All Fields] OR “alaska native*”[All Fields] OR “oceanians”[MeSH Terms] OR “maori people”[MeSH Terms] OR “maori*”[All Fields]) AND (((“pregnancy”[MeSH Terms] OR “pregnan*”[All Fields] OR “pregnant people”[MeSH Terms] OR “prenatal”[All Fields] OR “perinatal”[All Fields] OR “postnatal”[All Fields] OR “prenatal care”[MeSH Terms]) AND “perinatal care”[MeSH Terms]) OR “postnatal care”[MeSH Terms] OR (“antenatal”[All Fields] OR “antenatally”[All Fields]) OR “postpartum period”[MeSH Terms] OR (“postpartum period”[MeSH Terms] OR (“postpartum”[All Fields] AND “period”[All Fields]) OR “postpartum period”[All Fields] OR “postpartum”[All Fields]) OR “parturition”[MeSH Terms] OR “childbirth”[All Fields] OR “birthing”[All Fields])) AND (“exercise”[MeSH Terms] OR “exercise”[All Fields] OR “exercises”[All Fields] OR “exercise therapy”[MeSH Terms] OR (“exercise”[All Fields] OR “therapy”[All Fields]) OR “exercise therapy”[All Fields] OR “exercising”[All Fields] OR “exercise s”[All Fields] OR “exercised”[All Fields] OR “exerciser”[All Fields] OR “exercisers”[All Fields] OR “aerobic exercise”[All Fields] OR (“motor activity”[MeSH Terms] OR (“motor”[All Fields] AND “activity”[All Fields]) OR “motor activity”[All Fields]) OR “physical activity”[All Fields] OR “health promotion”[MeSH Terms] OR (“health”[All Fields] AND “promotion”[All Fields]) OR “health promotion”[All Fields] OR “smoking cessation”[MeSH Terms] OR (“tobacco use”[MeSH Terms] OR “tobacco”[All Fields] OR “tobacco use”[All Fields]) OR “nutrition”[All Fields] OR “nutritional status”[MeSH Terms] OR “diet, food, and nutrition”[MeSH Terms] OR “alcohol drinking”[MeSH Terms] OR “social support”[MeSH Terms] OR “well-being”[All Fields] OR “mental health”[All Fields] OR “social health”[All Fields] OR “mental health”[MeSH Terms])	January 2025–May 2025

## Appendix B. MMAT (2018) Appraisal of Included Peer-Reviewed Studies

**Author**	**Criteria Set**	**Methodological Quality Criteria 1**	**Methodological Quality Criteria 2**	**Methodological Quality Criteria 3**	**Methodological Quality Criteria 4**	**Methodological Quality Criteria 5**
Gould et al. (2022) [33]	Mixed methods	Yes	Yes	Yes	Yes	No
Barlow et al. (2015) [24]	Quantitative RCT	Yes	Can’t tell	Yes	Yes	Yes
Campbell et al. (2018) [34]	Qualitative	Yes	Yes	Yes	Yes	Yes
Eades et al. (2013) [35]	Quantitative RCT	Yes	Can’t tell	No	Can’t tell	Can’t tell
Patten et al. (2020) [23]	Quantitative RCT	Yes	No	Can’t tell	No	No
Gould et al. (2019) [37]	Mixed methods	Yes	Yes	Yes	Yes	Yes
Glover et al. (2016) [20]	Quantitative descriptive	Yes	Can’t tell	Yes	No	Yes
Patten et al. (2010) [22]	Quantitative RCT	Can’t tell	Yes	Yes	No	Yes
Lowell et al. (2015) [38]	Qualitative	Yes	Yes	Yes	Yes	Yes
CIRCA (2025) [39]	Mixed methods	Yes	Yes	Yes	Can’t tell	Yes

## Appendix C. Detailed Characteristics of Included Programs and Sources

**Reference**	**Program Name/Intervention**	**Location**	**Indigenous Population**	**Setting**	**Target Participants**
Winnunga Nimmityjah Aboriginal Health and Community Services [54]	Womens Group	ACT, Australia	Aboriginal and Torres Strait Islander	ACCHO/ACCHs	Pregnant women
Winnunga Nimmityjah Aboriginal Health and Community Services [54]	Healthy Cooking Group	ACT, Australia	Aboriginal and Torres Strait Islander	ACCHO/ACCHs	Entire community
Winnunga Nimmityjah Aboriginal Health and Community Services [54]	Pregnancy Group	ACT, Australia	Aboriginal and Torres Strait Islander	ACCHO/ACCHs	Pregnant women
Armajun Aboriginal Health Service [55]	Armidale Mums and Bubs	NSW, Australia	Aboriginal and Torres Strait Islander	ACCHO/ACCHs	Women
Biripi Aboriginal Corporation [41]	Child and Family Health Program	NSW, Australia	Aboriginal and Torres Strait Islander	ACCHO/ACCHs	Women
Bullinah Aboriginal Health Service [56]	Mums and Bubs Yarn Up Program	NSW, Australia	Aboriginal and Torres Strait Islander	ACCHO/ACCHs	Women
Durri Aboriginal Corporation Medical Service [57]	Aboriginal Maternal and Infant Health Services	NSW, Australia	Aboriginal and Torres Strait Islander	ACCHO/ACCHs	Women
Wurli Wurlinjang Aboriginal Corporation [43]	Tackling Indigenous Smoking (TIS) Program	NT, Australia	Aboriginal and Torres Strait Islander	ACCHO/ACCHs	Entire community
Nganampa Health Council Incorporated [47]	Tackling Indigenous Smoking (TIS) Program—Tjikita—Nyuntu Ngayuku Malpa Wiya	SA, Australia	Aboriginal and Torres Strait Islander	ACCHO/ACCHs	Entire community
Tullawon Health Service Incorporated [48]	Child and Maternal Health Program	SA, Australia	Aboriginal and Torres Strait Islander	ACCHO/ACCHs	Women
Beagle Bay Community Incorporated (KAMS Clinic) [40]	Australian Family Partnership Program	WA, Australia	Aboriginal and Torres Strait Islander	ACCHO/ACCHs	Women, significant others
Beagle Bay Community Incorporated (KAMS Clinic) [40]	Tackling Indigenous Smoking (TIS) Program	WA, Australia	Aboriginal and Torres Strait Islander	ACCHO/ACCHs	Entire community
Derbarl Yerrigan Health Service Aboriginal Corporation [42]	Maternal and Child Health Program	WA, Australia	Aboriginal and Torres Strait Islander	ACCHO/ACCHs	Pregnant women
Ngangganawili Aboriginal Community Controlled Health & Medical [53]	Maternal and Child Health Program	WA, Australia	Aboriginal and Torres Strait Islander	ACCHO/ACCHs	Pregnant women
Nindilingarri Cultural Health Service Incorporated [46]	Mums, Bubs and Strong Families Health Promotion Program	WA, Australia	Aboriginal and Torres Strait Islander	ACCHO/ACCHs	Women, significant others
Ord Valley Aboriginal Health Services Aboriginal Corporation (member of KAMS) [49]	The Fetal Alcohol Spectrum Disorder (FASD) team	WA, Australia	Aboriginal and Torres Strait Islander	ACCHO/ACCHs	Pregnant women
Kirrae Health Services Incorporated [45]	Deadly Walkers	Vic, Australia	Aboriginal and Torres Strait Islander	ACCHO/ACCHs	Pregnant women, entire community
Aboriginal and Torres Strait Islander Community Health Service Brisbane Limited [50]	Deadly Fit Mums	QLD, Australia	Aboriginal and Torres Strait Islander	ACCHO/ACCHs	Women in the perinatal period
Carbal Aboriginal and Torres Strait Islander Health Services Ltd. [51]	Strong born Mums group	QLD, Australia	Aboriginal and Torres Strait Islander	ACCHO/ACCHs	Pregnant women
Institute for Urban Indigenous Health Ltd. [52]	Deadly Fit Mums	QLD, Australia	Aboriginal and Torres Strait Islander	ACCHO/ACCHs	Women in the perinatal period
Townsville Aboriginal and Torres Strait Islander Corporation for Health Services [58]	The Maternal and Child Health team	QLD, Australia	Aboriginal and Torres Strait Islander	ACCHO/ACCHs	Pregnant women
Yulu-Burri-Ba Aboriginal Corporation for Community Health [44]	Deadly Fit Mums	QLD, Australia	Aboriginal and Torres Strait Islander	ACCHO/ACCHs	Women in the perinatal period
Gould et al. (2022) [33]	SISTAQUIT	NSW, SA, QLD, Australia	Aboriginal and Torres Strait Islander	ACCHO/ACCHs	Health practitioners
Askew et al. (2019) [2]	Smoking-cessation health promotion intervention	QLD, Australia	Aboriginal and Torres Strait Islander	ACCHO/ACCHs	Pregnant women, significant others
Barlow et al. (2015) [24]	Family Spirit Program	US	Native American	Community Outreach	Teen mothers
Campbell et al. (2018) [34]	Baby One Program	QLD, Australia	Aboriginal and Torres Strait Islander	Community Outreach	Women, families, health practitioners
Eades et al. (2013) [35]	Smoking-cessation health promotion intervention	QLD, WA, Australia	Aboriginal and Torres Strait Islander	ACCHO/ACCHs	Pregnant women
Patten et al. (2020) [23]	Healthy Pregnancies	Alaska, US	Alaska Native	Community Outreach	Pregnant women
Bar-Zeev et al. (2017) [36]	ICANQUIT (Protocol)	NSW, QLD, SA	Aboriginal and Torres Strait Islander	ACCHO/ACCHs	Health practitioners
Gould et al. (2019) [37]	ICANQUIT (Full study)	NSW, QLD, SA	Aboriginal and Torres Strait Islander	ACCHO/ACCHs	Pregnant women, health practitioners
Glover et al. (2016) [20]	“Aunties” cessation program	New Zealand	Māori	Community Outreach	Pregnant women
Patten et al. (2010) [22]	Smoking-cessation health promotion intervention	Alaska, US	Alaska Native	Community Outreach	Pregnant women
Lowell et al. (2015) [38]	Strong women, strong babies, strong culture program	NT, Australia	Aboriginal and Torres Strait Islander	Community Outreach	Health practitioners, community members
